# Feasibility and usability of a wearable sensor system for gait assessment in children with neuromuscular diseases

**DOI:** 10.3389/fresc.2025.1719215

**Published:** 2026-01-15

**Authors:** Nicoletta Battisti, Maria Giovanna Verrengia, Milena Pagnoni, Nadia Sommella, Sabato Mellone, Silvia Orlandi, Antonella Cersosimo

**Affiliations:** 1 Operative Unit Pediatric Rehabilitation Medicine, IRCCS Istituto delle Scienze Neurologiche di Bologna (ISNB), Bologna, Italy; 2 Rehabilitation Bioengineering Lab, IRCCS Istituto delle Scienze Neurologiche di Bologna, Bologna, Italy; 3 Department of Technical and Rehabilitation Assistance (DATeR), ASL Bologna, Bologna, Italy; 4 Department of Electrical, Electronic and Information Engineering “Guglielmo Marconi” (DEI), University of Bologna, Bologna, Italy; 5 Department of Electrical, Electronic and Information Engineering “Guglielmo Marconi” (DEI), Health Sciences and Technologies, Interdepartmental Center for Industrial Research (CIRI-SDV), University of Bologna, Bologna, Italy

**Keywords:** gait, gait analysis, movement, neuromuscular diseases, sensors, walking, wearable devices

## Abstract

**Introduction:**

In children with Neuromuscular diseases (NMDs), gait monitoring is essential for evaluating motor function over time. The 10-meter walk test (10 mWT) and the 6 Minute Walking test (6 MWT) are commonly used timed tests. Wearable inertial measurement units (IMUs) have recently gained increasing interest and use for gait assessment. The primary objective of the study is to verify the technical feasibility and clinical usability of IMUs in children with NMDs during standard 10 and 6 MWT. Secondly, the authors aimed to investigate the agreement between the results of manual and device-based tests and the device's measurements of the 95th percentile of stride speed (SV95C) and its correlation with the 6 MWT.

**Methods:**

Ambulatory children aged 6–18 with NMDs were enrolled. The IMU used for the study was the mTest^3^® device. The 10 mWT and 6-MWT were performed both in the standard method and with the device. Feasibility was assessed through the completion rates of tests with the device, the thematic analysis of clinicians’ feedback, timing for the device's use and the percentage of usable recordings. Usability was evaluated using the System Usability Scale (SUS) questionnaire and a pediatric-adapted semi-structured patient interview. Agreement between the results of manual and device-based tests and the device's measurements of the 95th percentile of stride speed (SV95C) and its correlation with the 6 MWT were also evaluated.

**Results:**

Twelve patients with NMDs and a mean age of 12 years and 9 months (range: 8–17 years) were enrolled. All patients completed the assessment protocol using the device. Feedback from clinicians was positive, with few outlier recordings identified. Results from the SUS questionnaire and patient interview showed predominance of positive judgments. The study showed good agreement between the measurements, particularly for the 6 MWT. High correlation between SV95C and 6 MWT was also demonstrated.

**Conclusion:**

This study confirmed the feasibility and usability of IMUs for gait assessment in children with NMDs. In addition, agreement between device-based and manual-based measurements and high correlation between SV95C and 6 MWT was also demonstrated. IMUs can serve both as clinical outcome assessment tools and as devices for gait monitoring across various contexts, supporting their integration into pediatric rehabilitation protocols.

## 1 Introduction

Neuromuscular diseases (NMDs) constitute a broad and heterogeneous spectrum of rare, acquired or congenital-hereditary disorders causing structural and functional alterations of the motor unit (1). These disorders progressively impair motor performance and often lead to substantial functional limitations. The worldwide prevalence of NMDs is estimated at 48 out of 100'000 individuals annually (2). The Duchenne Muscular Dystrophy (DMD) represents the most common childhood-onset NMD, with a reported worldwide prevalence of 2.8 per 100,000 inhabitants (3). Pediatric-onset NMDs are characterized by progressive clinical deterioration over time (4). Walking is one of the motor functions that is constantly compromised and it is also an important marker of disease progression. The gradual reduction of walking speed and travelling distance reflects the degree of worsening of the disability (4). Preserving independent walking is a primary therapeutic objective in these conditions. Gait monitoring is also essential for evaluating treatment effectiveness and motor function over time. Several validated timed tests are used to assess and monitor functional gait changes in NMDs (5). The 6 Minute Walking Test (6 MWT) (6–13), which measures the distance covered in 6 min, is widely used in clinical practice and in pharmacological trials as a functional endpoint. Similarly, the 10-Meter Walk Test (10 mWT) is also frequently used in clinical practice for ambulatory patients (14–16). The 10 mWT measures walking speed over a 10-meter distance. While these tests provide valuable global metrics of motor performance, they do not capture the underlying spatio-temporal gait features that might reveal early or subtle functional changes. Recent advances in wearable technology have led to a growing interest in portable gait analysis systems that enable quantitative assessment outside of specialized motion laboratories. Conventional gait laboratories, based on stereophotogrammetric systems and force platforms, continue to represent the gold standard for biomechanical evaluation; however, their implementation is limited by cost, the necessary expertise, and restricted ecological validity (17, 18). During the last few years, many portable solutions have been proposed for the assessment and monitoring of movement disorders and gait (19). Among these, wearable inertial measurement units (IMUs) have gained increasing interest and use (20). IMUs are systems applicable to different parts of the body, inexpensive and easy to use, which allow monitoring of activity levels in different contexts and have demonstrated high reliability as measuring instruments (21). They can be used alone or with other sensors to monitor motor performance and rehabilitation (22). IMUs evaluate different kinematic parameters of gait through the measurement and processing of acceleration and angular velocity using specific algorithms and these parameters have the potential to be technological outcome measures (23–26). In this context, integrating IMUs into clinical gait tests offers an opportunity to obtain quantitative spatio-temporal parameters that are not captured by conventional timed assessments (20). Traditional tools–such as questionnaires, motor scales, and timed tests—provide global clinical outcomes (e.g., distance walked or time to complete a task) but lack the capacity needed to detect subtle gait alterations and early functional decline. IMUs complement these measures by extracting objective parameters (e.g., stride length, cadence, variability, and maximal stride velocity) during clinical tests, without modifying their clinical setup (27). This simultaneous acquisition may increase the sensitivity and precision of outcome measures, enhance validity by assessing gait performance in routine clinical conditions, and reduce the need for specialized laboratory equipment or personnel. As a result, IMU-based metrics have the potential to support clinicians in decision-making, streamline clinical workflows, and provide more responsive markers of disease progression and treatment effects (28–30). Some of these parameters, such as the 95th percentile of stride length, median stride speed, and especially the 95th percentile of stride speed (SV95C) are measures which are sensitive to changes in patients with DMD (23–26). The SV95C represents 5% of the fastest steps and reflects maximal motor performance while walking. The SV95C has been shown to have a strong correlation with the results of the 6 MWT (26). The SV95C has also been included as a digital endpoint by the European Medicine Agency (23–25) for gait monitoring in DMD. Most of the IMUs require placement on multiple body segments or joints (such as feet, ankles, and trunk), and, despite of the lack of standardization (31), this choice could depend on the type of motor task to analyze. For example, some studies showed higher accuracy of gait pattern analysis if wearable sensors were placed on segments proximal to the feet, and maximizing the performance when they are directly placed on the shoes (32). Conversely, some other IMUs are incorporated into wearable equipment, such as pressure insoles (32). Comfort and lightweight sensors are essential characteristics for ensuring users’ acceptance (33, 34) and reliability in capturing gait patterns. These elements, together with higher power consumption and reduced robustness (35) limit the use of shoe insoles, although they offer potential to be completely integrated into practical use. Despite these emerging advantages, IMU-based gait analysis in pediatric NMDs remains insufficiently explored. One reason may be found in the rarity of these diseases; another reason may be attributed to the variability of gait patterns among different disorders, influencing the IMUs-based analysis outcomes, which are rarely validated in abnormal conditions (21). Other reasons are, on the other hand, more strictly linked to age, which strongly influences the variability of gait pattern (36). This represents a significant gap in the literature, as NMDs often demonstrate progressive gait impairments requiring frequent and sensitive monitoring.

Given the lack of documented applications in the literature of this technology, specifically concerning the positioning of the sensor element and the utilization of the device's hardware and software interfaces by clinical operators, the aim of this study is to verify the technical feasibility and clinical usability of the IMU-based device for gait assessment in children with NMDs during the 10 and 6 MWT in clinical practice. IMU sensors can improve the precision of these tests in clinical contexts and make their administration easier for operators. The standard 10 and 6 MWT require a complex set-up and involve the manual calculations of walking speed and distance covered by operators. This procedure may lead to errors and is time-consuming. The feasibility analysis assesses the practicality and user-friendliness of the device, providing justification for its implementation in clinical practice. The usability analysis evaluates the device's effectiveness, efficiency, and satisfaction for both operators and patients. A further analysis of the agreement between the manual and device-based measurements is also conducted. Additionally, the study explored the device's potential to measure the SV95C and its correlation with conventional functional outcomes such as the 6 MWT.

## 2 Methods

### 2.1 Study design and ethical approval

This prospective monocentric interventional post-market study was conducted at the Operative Unit of Pediatric Rehabilitation Medicine, IRCCS of Neurological Sciences of Bologna, Italy in collaboration with the Department of Technical and Rehabilitation Assistance (DATeR) of AUSL Bologna, Italy the Department of the University of Bologna (DEI), and the Laboratory of Rehabilitation Bioengineering of IRCCS of Neurological Sciences of Bologna, Italy. The study complied with the Declaration of Helsinki and received AVEC Local Ethics Committee approval (ref. CE AVEC number 520-2023-DISP-AUSLBO—SIRER ID 6309).

### 2.2 Participants

Ambulatory children aged 6–18 with a confirmed diagnosis of NMDs were consecutively enrolled. Participants were required to possess sufficient cognitive abilities to understand and follow testing instructions. Exclusion criteria comprised: (i) current or previous use of orthoses or assistive devices during ambulation, and (ii) need for supervision due to a clinically identified risk of falls. All enrolled individuals were independent walkers at the time of assessment. Written informed consent was obtained from all participants and their parents.

### 2.3 Instrumentation

**Figure 1:**
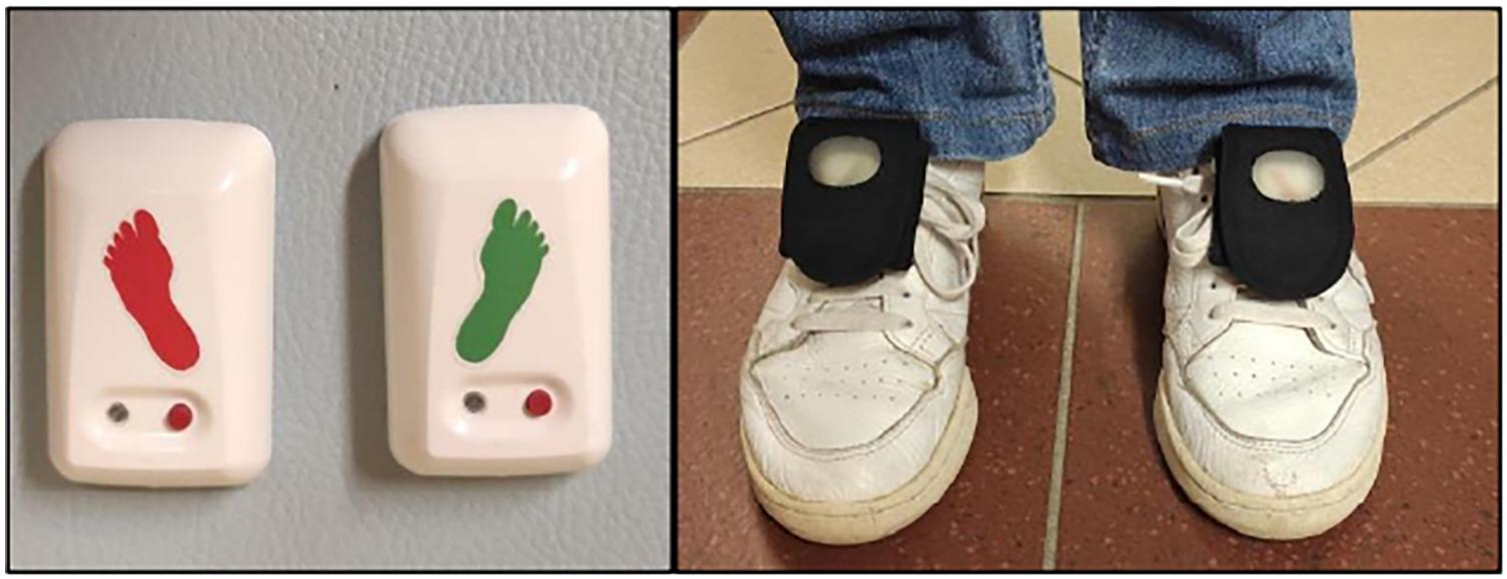
The sensors (right side in green and left side in red) are inserted with the foot image facing down into a velcro pocket that is attached to the shoe laces.

Gait assessment was performed using the mTest^3^® (mHealth Technologies s.r.l., Monte San Pietro, Bologna, Italy), a certified wearable medical device comprising two inertial measurement units attached to the shoelaces (Figure 1). The device includes three assessment modules implemented as separate smartphone applications: *mGait*, for the gait analysis, *mTUG*, to perform the Time-Up-and-Go Test, and *mSway* for the balance assessment. The *mGait* enables the administration and automated processing of standard walking tests, including the 10 mWT and the 6 MWT, through two inertial sensors placed on the shoelaces. The device-based test scores adopted were the median gait speed and the distance covered respectively, calculated by the algorithm itself. This device has been tested both on adult patients with normal pressure hydrocephalus and Parkinson's (26), and in children with Cerebral Palsy (37), but never in pediatric patients with NMDs. The *mGait* embeds an algorithm for the assessment of spatio-temporal gait parameters (38–40)—e.g., stride length, cadence, gait speed, stride duration, pitch contact. These parameters, computed step-by-step, are reported in a *.csv* file, while their distributions were presented through box-plots and median values in a report for the user. Lanovaz (41) observed that the stride length estimation tends to overestimate short strides and underestimate long ones, particularly in pediatric populations. To address this bias, during this study the mTest^3^® algorithm was preliminary tested and appropriately adapted. The thresholds for determining the zero velocity update in the algorithm for the gait analysis (39), were modified to take into account that, in the target population, the foot contact could also be only with the toe, and that the foot might not be completely still. Prior to data collection, clinicians participating in the study completed standardized training and were required to conduct at least five independent sessions using the device to ensure adequate familiarization.

### 2.4 Procedures

All participants underwent a baseline clinical evaluation, including joint range of motion assessment, muscle strength estimation, and administration of the MFM-32 scale (42) to quantify functional impairment. Each patient completed two 10 mWT trials and one 6 MWT trial, with a 10-minute rest between sessions. The timed motor function tests were explained to the children using the instruction: “*Walk without running, as you walk every day*.” According to standard test recommendations (9, 15, 16), a distance of 25 m was set up for the 6 MWT and 10 m for the 10 MWT. The 6 MWT test area was marked with a 25-m tape line. The tape line was placed in the middle of the corridor and marked at 1-m intervals. A cone was positioned at each end of the course. The participants performed the 10 MWT on a regular, flat surface, with the start and end points of the path clearly marked on the floor for the examiners. The tests were recorded by two operators contemporarily using the standard methodology and the mTest^3^® device. The standard and device-based 10 MWTs scores were calculated as the median gait speed by the operators and the algorithm itself respectively. Perceived exertion was recorded pre- and post-test using the Borg scale (43). Operator roles’ (details are provided in Supplementary Material) were randomized and blinded.

### 2.5 Feasibility, usability, and agreement assessment

Feasibility was assessed through the completion rates of tests using the device, the thematic analysis of clinicians’ feedback, timing for sensor placement, activation, and data export, and the percentage of usable recordings. Usability was evaluated according to ISO 9241-11 (44) using the System Usability Scale (SUS) questionnaire (45, 46) completed by clinicians, and a pediatric-adapted, semi-structured patient interview (consisting of an adaptation of the SUS questionnaire, using language more appropriate to the pediatric population). They both consisted of ten items with response options on a 5-point Likert scale, and an open field was added to collect possible comments. Agreement between manual and device measurements was analyzed for both tests. Possible associations between the 6 MWT score (both device-based and manually computed) and the SV95C were also investigated.

### 2.6 Statistical analysis

Due to the rarity of NMDs and potential drop-out risks, a sample of 12 patients was selected (47–50).

*Feasibility* was considered achieved if at least 80% of the judgments expressed by the three clinicians were positive and if at least 80% of the total recordings were usable. Outliers in both device and standard method scores distribution were identified using Tukey's test (51), for identification of potentially incorrect observations due to different causes such as the inaccuracy of the device or the incorrect positioning of the sensors.

*Usability* was considered satisfactory with SUS and patient interview scores ≥70 out of 100 or excellent with scores ≥85 out of 100 (52). The Bland–Altman plot (53) was used to assess the agreement between standard and device measurements in the two tests. Additionally, after having verified non-normal distribution of variables through the Shapiro–Wilk test, the Mann–Whithney-*U* test was adopted for detecting possible statistical differences between manual-based and device-based score distribution. Spearman's correlation coefficient between the 6 MWT score and SV95C was calculated. Stride velocity was computed as the ratio between stride length and stride duration, and the SV95C was computed as the 95th centile of the stride velocity distribution (54).

## 3 Results

Twelve patients (2 females) with a mean age of 12 years and 9 months (range: 8–17 years) were enrolled in the study. Table 1 reports patients’ diagnoses, anthropometric characteristics, and MFM-32 scores. All patients were ambulatory without assistive devices and did not present significant joint limitations or muscle strength deficits. The mean MFM-32 total score was 84.71% (range: 57.29%–100%), indicating a moderate degree of functional impairment (42).

**Table 1:** Diagnosis and anthropometric data of enrolled patients: age and degree of functional impairment measured using the MFM-32 scale.

Patient	Pathology	Sex	Age	MFM-32 scale	Weight (in kg)	Height (in m)	BMI
P001	Central core myopathy	M	15 years	89.58%	67	1.72	22.7
P002	Duchennedystrophy	M	8 years	91.70%	22	1.19	15.53
P003	Steinert dystrophy	M	10 years	57.29%	19.8	1.24	12.9
P004	Steinert dystrophy	F	17 years	72.91%	54	1.53	23
P005	Duchennedystrophy	M	11 years	78.00%	22	1.32	12.64
P006	Spinal muscular atrophy 3	M	9 years	94.79%	35	1.25	22.43
P007	Duchennedystrophy	M	15 years	94.79%	35	1.35	19.2
P008	Calpainopathy	M	15 years	71.87%	85	1.80	26.2
P009	Spinal muscular atrophy 3	M	8 years	84.37%	22	1.28	13.42
P010	Selenopathy	M	16 years	91.66%	41.5	1.75	13.55
P011	Steinert dystrophy	M	15 years	100.00%	45	1.63	16.9
P012	Merosin deficit dystrophy	F	16 years	89.58%	45	1.62	17.1

Table 2 summarizes the results of the tests performed using the device, alongside the outcomes of the standard 10 and 6 MWT. In the first trial of the standard 10 mWT, the mean walking speed was 1.31 m/s (range: 0.82–2.58 m/s), which remained substantially unchanged in the second trial at 1.30 m/s (range: 0.83–2.61 m/s). The average walking speed recorded with the device was lower: 1.10 m/s (range: 0.73–1.91 m/s) in the first trial and 1.19 m/s (0.73–2.08 m/s) in the second. In the standard 6 MWT, the mean distance covered was 365.05 m (range: 243–589 m), while the distance recorded with the device was comparable, averaging 363.95 m (range: 229–564 m). The differences were not statistically significant.

**Table 2:** Standard and mTest^3^-based 10 mWT and 6 MWT scores: speed (m/s) and distances covered in the 10 mWT and 6 MWT.

ID	Standard			mTest^3^		
	10 mWT		6 MWT	10 mWT		6 MWT
	T1	T2		T1	T2	
P001	1.47 m/s	1.27 m/s	502.8 m	1.40 m/s	1.40 m/s	521.61 m
P002	1.45 m/s	1.19 m/s	302.5 m	1.26 m/s	1.10 m/s	304.26 m
P003	0.85 m/s	0.85 m/s	376.6 m	0.82 m/s	0.86 m/s	373.28 m
P004	1.12 m/s	1.19 m/s	325.4 m	1.00 m/s	1.10 m/s	324.82 m
P005	0.82 m/s	0.83 m/s	252.5 m	0.73 m/s	0.73 m/s	261.39 m
P006	1.49 m/s	1.80 m/s	478.2 m	1.45 m/s	1.73 m/s	454.45 m
P007	1.18 m/s	1.19 m/s	389.0 m	1.08 m/s	1.09 m/s	382.22 m
P008	0.84 m/s	0.93 m/s	247.0 m	0.83 m/s	0.87 m/s	264.29 m
P009	1.18 m/s	1.19 m/s	259.0 m	1.09 m/s	0.95 m/s	255.15 m
P010	2.58 m/s	2.61 m/s	589.0 m	1.91 m/s	2.08 m/s	564.04 m
P011	1.47 m/s	1.20 m/s	414.5 m	1.29 m/s	1.18 m/s	432.25 m
P012	1.35 m/s	1.35 m/s	243.8 m	1.17 m/s	1.19 m/s	229.71 m
Tukey's test result	(0.43, 2.10)	(0.87, 1.53)	(−3.76, 690.94)	(0.41; 1.85)	(0.47, 1.70)	(−3.42, 606.75)

m, meters; s, seconds; T1, Trial 1; T2, Trial 2.

### 3.1 Feasibility

All recruited patients completed the trials using the device, achieving 100% protocol adherence. Analysis of clinicians feedback, collected via a dedicated open section, highlighted the following issues: in eight cases, difficulties related to available spaces, in three cases, problems with the connection and synchronization of the device; in two cases, failure to update the date and time on the smartphone; and in one case, difficulty in securing the device to footwear equipped with velcro instead of laces. The mean time required to apply the device was 5 min and 27 s (don: 2′55″, doff: 2′31″). All clinicians recruited in the study, responded favorably to the possibility of using the mTest^3^® by a single clinician in clinical practice, asserting “*ease of use*” as the main motivation. Out of 36 total registrations, 35 (97.22%) were deemed valid. Outliers’ identification was conducted using Tukey's method, during both the 10 mWT and 6 MWT (standard and device-based version). The number of outliers was greater in the standard 10 mWT (*n* = 5) than device based 10 mWT (*n* = 2), while no outliers were detected in either method for the 6 MWT.

### 3.2 Usability

**Figure 2:**
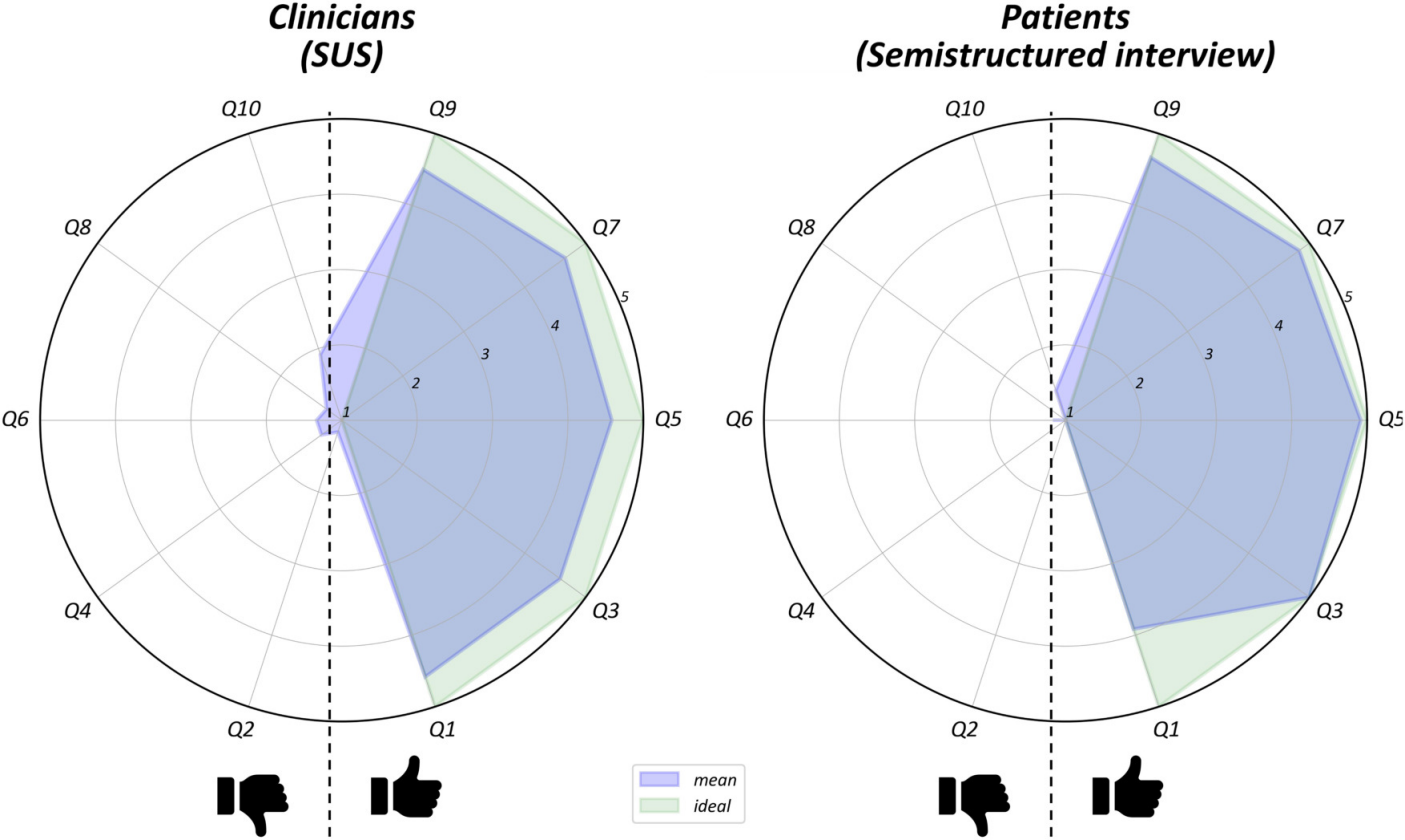
Item-by-item results of clinicians SUS questionnaire and patients semi-structured interview. They bothconsisted of ten items with response options on a 5-point Likert scale. For each radar plot items with positive (on the right) are split from those with negative (on the left) meaning. The green area indicates the hypothetical maximal appreciation of the device, while the blue area the mean of users judgments.

Results from the SUS questionnaire and the semi-structured interview administered to clinicians and patients showed predominance of positive judgments with respect to the usability of the device. The overall average score for operators (Figure 2) was 89.79 (range: 83.75–98.125), showing excellent usability values. Similarly, the semi-structured interviews administered to patients (Figure 2) yielded a high mean usability rating (94.4). Three patients completed section 2 of the interview: two expressed a preference for smaller sensors, while one patient reported a positive opinion about participating in the study (“*I had fun*”).

### 3.3 Agreement and correlation analysis

**Figure 3:**
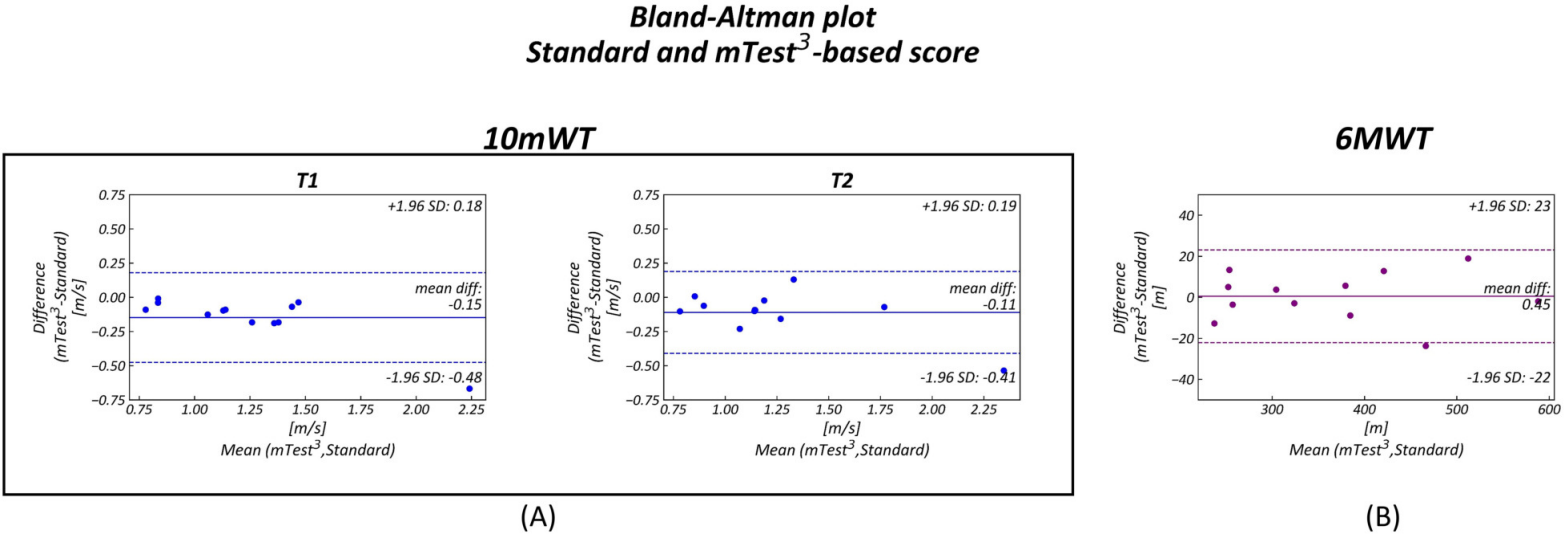
Bland–Altman Plots comparing standard and device-based (mTest^3^) measurements. Each plot shows on the *y*-axis the difference between the standard and the mTEST^3^ measurements and on the *x*-axis the mean of them. The limits of agreement are represented as dotted lines (±1.96 SD) while mean differences are displayed as continuous lines. In all three Bland–Altman plots the distributions fall inside the limits of agreement. **(A)** 10 MWT first (T1) and second (T2) trial **(B)** 6 MWT.

Bland–Altman plots (Figure 3) demonstrated good agreement between the measurements, particularly for the 6 MWT. Although in the 10 mWT the results were slightly lower, all but one data point fell within the limits of agreement, and the mean differences approached zero, with values contained within narrow confidence intervals.

The correlation coefficients between SV95C and standard 6 MWT were 0.92 (*p* < 0.001) and 0.91 (*p* < 0.001) for the device-based measurements, both indicating a highly significant relationship.

## 4 Discussion

In recent years, IMUs have progressively expanded in several fields (55), thanks to increasing advantages deriving from miniaturization (30). In rehabilitation, they show great potential for obtaining more precise information in clinical settings as well as ecological ones. Few previous research activities seeking the feasibility of using wearable devices were conducted on children. The most adopted sensor system across the majority of the studies was Mobility Lab (APDM, Inc., Portland, OR) proven to be useful and accurate in the movement pattern analysis of various adult neurological patients (56–58). A lower number of studies investigated the feasibility of using IMUs in children, showing an adherence to the protocols of almost 80% (59). In our study, adherence to the protocol was higher and the number of outliers low, highlighting the good feasibility of the device in children. In his review, Fonseca (31) showed that wearable sensors can be adopted for the evaluation of children affected by different pathological conditions. Several studies in the literature have shown the use of body-worn inertial sensors for investigating the gait pattern abnormalities in children with cerebral palsy (60). Ganea in 2012 (61) recruited individuals with DMD, while most of the studies focused on Spina bifida (58, 62), Cerebral palsy (37, 60, 63–65), and healthy individuals (24, 66, 67). This confirms the scarcity of literature about neuromuscular pediatric conditions (68). Our study provides evidence that wearable inertial measurement technology can be feasibly integrated into the clinical gait assessment of children with NMDs. The high completion rate, minimal technical difficulties, and favorable usability scores collectively demonstrate that the device can be readily implemented in routine settings without imposing an additional burden on clinicians or patients. These findings align with the increasing emphasis on digital mobility outcomes as complementary tools for neuromuscular disease monitoring. Studies on the use of IMUs as technological biomarkers for outcome assessment in rehabilitation have recently become increasingly widespread, thanks to the advances offered by these technologies. IMUs are used to assess and monitor motor function in order to improve the sensitivity of treatment outcome measures and functional assessments in different contexts and activities. Questionnaires, clinical motor scales, and timed tests constitute traditional psychometric tools commonly used in different clinical contexts, but they present a lack of objectivity (21). Bisi et al. (69) showed agreement between the IMU-based and manual-based outcome scores related to movement competence. Our study confirms the agreement between manual and device-based measurements with a potential role in clinical practice for gait monitoring. A key contribution of the study is the demonstration of good agreement between device-based and standard clinical metrics. These findings support the reliability of inertial sensor-derived measures during structured gait tasks that are well established in neuromuscular research. These devices offer the potential to evaluate and monitor motor functions with greater precision than traditional clinical protocols. In our study, the number of outliers was greater in the standard 10 mWT (*n* = 5) than device-based 10 mWT (*n* = 2), while no outliers were detected in either method for the 6 MWT. Despite the reduced sample size, the lower number of outliers in device-based measures may suggest that sensor-derived outcomes offer greater consistency. Other studies conducted on children with DMD confirm not only the feasibility of using the IMU in the pediatric population with DMD, but also the suitability and reliability of detected outcomes in assessing motor dysfunction (70). These gait abnormalities are important factors to be considered not only at the diagnosis, but also in the choice of treatment (60) and for evaluating the rehabilitation outcomes (61). Some parameters, such as the SV95C, have been shown to be measurements sensitive to changes in patients with DMD for monitoring activity levels in ecological contexts (23–26). The SV95C has been shown to have a strong correlation with the 6 MWT changes (26). In our study, we also confirmed a significant correlation between the SV95C and the 6 MWT. This result may be useful in future clinical studies for monitoring gait worsening in NMDs.

In summary, by confirming feasibility, usability, and alignment with established gait metrics, this preliminary study supports the incorporation of wearable inertial sensor technology into pediatric NMD gait assessment protocols. The results underscore its potential to expand current evaluation frameworks towards more objective, continuous, and ecologically valid monitoring paradigms.

### 4.1 Study limitations

This study presents some limitations that should be considered when interpreting the results. Firstly, the small sample size justified by the preliminary nature of the study and the rarity of pediatric neuromuscular diseases. Future studies should involve larger, multicenter cohorts to confirm the feasibility and usability of the device in different clinical settings. Moreover, the study exclusively focused on ambulatory patients with adequate cognitive abilities, excluding those with more severe disabilities or requiring walking aids. This restricts the clinical applicability of the device and prevents assessment of its potential utility in patients with higher functional impairment. From a technological perspective, the use of a single smartphone model and operating system may have influenced the reliability of connections and data management. Future research should test the device with a wider range of devices to evaluate its compatibility and performance in different technological configurations. Another limitation concerns the exclusive clinical setting. Although the device was designed for use in ecological and daily life contexts, this application has not yet been fully explored. Future studies should investigate the feasibility, usability, and patient adherence to device use in home and school environments, to verify its potential for continuous monitoring. Future directions also include the implementation of longitudinal analyses to assess the device's sensitivity in detecting functional changes over time and in response to pharmacological or rehabilitative interventions. Additionally, the development of dedicated algorithms tailored to specific pediatric NMDs phenotypes and the evaluation of predictive parameters such as the SV95C for clinically meaningful events, such as the loss of independent ambulation, are warranted. The adjustment of the thresholds for the gait analysis algorithm was empirical and only based on the small, non-representative sample of this feasibility study. It will be necessary to verify the adaptation on a larger, more representative sample before the algorithm update can be generalized and considered definitive. These results should, therefore, be regarded as indicative of the fact that it is possible to successfully adapt the method to the specific characteristics of gait in this target population, but are not in themselves sufficient to validate the modified version of the algorithm.

## 5 Conclusion

This study provides evidence that wearable inertial sensors can be effectively integrated into routine gait assessments in children with NMDs. Beyond demonstrating that the device can be used during standard clinical tests, these findings highlight the clinical relevance of obtaining objective spatio-temporal gait parameters without altering existing assessment protocols. The positive feedback from clinicians and patients confirms that the introduction of such technology does not add a substantial burden to the workflow, so addressing one of the primary barriers to the adoption of digital tools in pediatric settings. Importantly, the close agreement between device-based and manual measurements reinforces the reliability of the instrument, while the strong association between SV95C and the 6 MWT supports the potential of sensor-derived metrics as complementary outcome measures capable of capturing aspects of gait performance not reflected by distance or time alone. Collectively, these observations indicate that mTest^3^-based systems can enhance the sensitivity of gait monitoring in NMDs, bridging the gap between traditional clinical tests and more detailed biomechanical evaluations.

These findings provide a foundation for broader implementation of wearable-based assessments and encourage future studies aimed at evaluating their longitudinal sensitivity and clinical utility in pediatric NMD care and research.

## Data Availability

The datasets presented in this study can be found in online repositories. The names of the repository/repositories and accession number(s) can be found below: the data associated with the paper are available after study publication on a data research repository (https://zenodo.org/).

## Sitography

1. The mTest3 web site: https://mhealthtechnologies.it/products-wearable-sensors-smartphone-apps/assessment-mtest3/.
