# Supplementary material for: Feasibility and usability of a wearable sensor system for gait assessment in children with neuromuscular diseases
Source: Front Rehabil Sci. 2026 Jan 15;6:1719215. doi: 10.3389/fresc.2025.1719215 (PMC12852440; doi:10.3389/fresc.2025.1719215)
Supplement: Supplementary file 1 [file Datasheet1.pdf]

Role A (RA)	Administration of the Borg scale to the patient, at the start and end of each test
	Recording of peripheral blood saturation values, heartbeats and respiratory rate
	Administration of the semi-structured patient interview
Role B (RB)	Start the stopwatch to measure the total time the device has been used.<br>Inserting the sensors on the patient's footwear<br>Turning on the smartphone<br>Entering the patient's randomization code<br>Sensor synchronization<br>Stop the stopwatch
	Execution of the 10mWT: selection of the test on the drop-down menu, then start recording at ma0 with end at ma8 (two repetitions)
	Running the 6MWT: selecting the test on the drop-down menu, then starting the test (the device stops automatically after 6 minutes detecting the distance traveled).
	Restarting the stopwatch<br>Removing Sensors from Footwear<br>Transferring data to your PC<br>Stop the stopwatch and time recording<br>Filling in the operator questionnaire.
Role C (RC)	Execution of the 10mWT: start of the stopwatch when the marker of 2 meters is reached and interruption when the marker of 8 meters is reached. The test wascarried out in tworepetitions
	Execution of the standard 6MWT: instructions to the patient before and during the test, detection of the number of revolutions and any pauses.

Supplemental 1 - The table shows the three operators roles: A, B, and C. The roles were assigned on a rotating basis to the three studio operators, following a blind randomization with respect to the researcher, keeping the frequency of each role constant for each operator.
